# The potential of fNIRS, EEG, and transcranial current stimulation to probe neural mechanisms of resistance training

**DOI:** 10.3389/fnhum.2023.1295993

**Published:** 2023-11-30

**Authors:** Stéphane Perrey

**Affiliations:** EuroMov Digital Health in Motion, Univ Montpellier, IMT Mines Ales, Montpellier, France

**Keywords:** neuroplasticity, monitoring, biofeedback, brain, force

## Introduction

Investigating the neural mechanisms underlying physical performance is a growing research focus in the field of sport neuroscience. Sport is more and more benefiting from and contributing to a greater awareness of concepts such as neuroplasticity (i.e., the structural and functional adaptations in specific brain and spinal circuits), and neuromodulation techniques (i.e., the application of low-level intensity currents to induce polarity-specific changes in neuronal excitability). Neuroplasticity is not widely comprehended in the field of strength and conditioning; nevertheless, it fundamentally influences how athletes move and perform in sports. Understanding the basic concepts of neuroplasticity can guide strength training, which is defined as resistance exercise resulting in an increase in force capacity. To perform multi-joint movements, the brain must coordinate with suitable muscle groups to execute timely the muscle contraction. Thus, strength training related to motor learning, necessitates complex inter- and intra-muscular coordination initiated in the motor cortex. Furthermore, strength training results in use-dependent plastic changes over time (known as long-term potentiation, Cooke and Bliss, 2006) in the central nervous system (CNS), particularly within the motor cortex (Hortobagyi et al., 2021).

It is widely accepted that strength training requires neural adaptations in the early stages of training (Sale, 1988; Hortobagyi et al., 2021). This assumption is underpinned by research showing that initial stages of training lead to considerable enhancements in force generation, without concomitant alterations in muscle mass (i.e., structural changes). Specifically, motor unit adaptations in muscle force generation occur in the first few weeks of training (Häkkinen et al., 1985). However, until recently, the literature on strength training has not conclusively identified the parts of the CNS that are most responsible for these adaptations. A recent primate study has shown that strength training-induced supraspinal changes through the reticulospinal tract are associated with changes in muscle performance (Glover and Baker, 2020). Recent meta-analyses (Siddique et al., 2020; Hortobagyi et al., 2021; Gómez-Feria et al., 2023) have highlighted a trend toward a simultaneous rise in corticospinal excitability and muscle strength coupled with a reduction in corticospinal inhibition after undertaking resistance training. However, it is important to note that this trend presents with a considerable degree of heterogeneity depending on the chosen training modality (Gómez-Feria et al., 2023). To date, given the paucity of research on the neural impacts of strength resistance training, it remains unclear how much strength training is required to generate substantial and lasting neural changes.

Hortobagyi et al. (2021) reviewed changes in neuroplasticity underlying the increases in force induced by strength training in various sites. Recent technological advances have provided substantial new evidence of such neural adaptations. Beyond the current knowledge of motoneuron firing patterns using high-density surface electromyography and decomposition (Del Vecchio et al., 2019), corticospinal excitability and intracortical inhibition adaptations have been investigated with the advent of transcranial magnetic stimulation measures (Kidgell et al., 2017). While it is often claimed that strength training produces favorable changes in brain plasticity with motor performance, it remains overlooked. Besides electrophysiological measures, cortical neuroimaging techniques with applicability on the field, such as functional near-infrared spectroscopy (fNIRS) and electroencephalography (EEG), can elucidate the role of the motor cortex and other cortical brain areas and networks in response to strength training. In terms of brain areas, the primary motor cortex (M1) is most closely associated with exercise performance due to its role in motor execution, but other motor control-related cortical areas (premotor cortex, PMC; supplementary motor area, SMA and inferior parietal cortex) have the potential to complete the puzzle of the sites of neural adaptations. Our understanding is restricted concerning the outcomes of strength training on brain regions and their cooperation as functional networks, underpinning increased motor functions. The unique cortical activation profiles linked with superior muscle performance could unveil neural benefits. This is in line with the neural efficiency hypothesis postulating that experts possess a more efficient cortical processing (fewer neural resources) than non-experts (Neubauer and Fink, 2009; Li and Smith, 2022).

**Figure 1:**
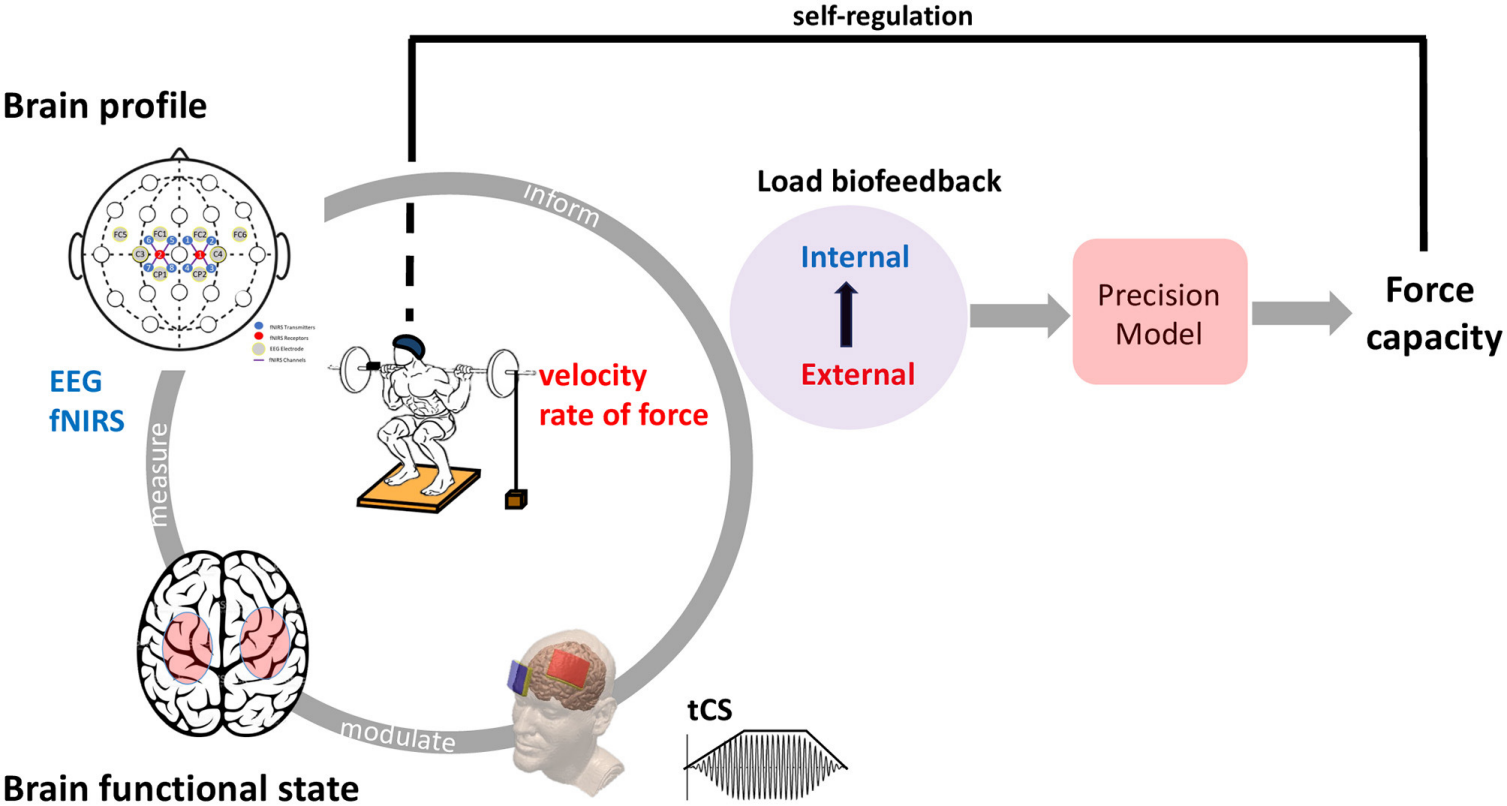
*Brain functional state:* Assessment of brain activity using a combination of electroencephalography (EEG) and functional near-infrared spectroscopy (fNIRS) signals in relation to resistance exercise movements and/or the impact of non-invasive transcranial current stimulation (tCS) on task-related neuronal activity levels. EEG records neuro-electrical signals related to cortical neuronal activity via scalp electrodes, whilst fNIRS measures dynamic fluctuations in the concentration of oxygenated blood flow via optodes. *Brain profile*: Combined EEG and fNIRS allow for non-invasive real-time monitoring of the neurometabolic status of various cortical motor brain areas during simple to complex movements in strength training. The illustrated layout enables investigation of the motor cortical regions over both hemispheres with 4 EEG electrodes (gray filled circles) and 4 fNIRS optodes (4 fNIRS transmitters -blue circles- and one receiver -red circles-) on each hemisphere. It is not possible to access deep brain structures using surface EEG and fNIRS. *Load biofeedback:* The precision strength training module is able to quantitatively measure and model both the internal (brain profile) and external (kinematics and kinetics) loads that individuals consider by a biofeedback approach, and propose adjustments to the training program based on changes in force capacity. The external load feedback delivers real-time performance data on the actual barbell velocity (linear velocity transducer) and/or rate of force development (orange force plate) achieved during each repetition of movement.

There is also great interest in sport in the development of methods to enhance muscle strength by modulating neural drive. In contrast to EEG-fNIRS techniques, which measure neural correlates of behavior, non-invasive brain stimulation (e.g., transcranial -direct or alternating- current stimulation, tCS) offers the possibility of perturbing neural information processing and measuring its effects on behavior. Indeed, tCS can be used as a neuromodulatory ergogenic resource for healthy individuals to induce neuroplasticity and increase muscle strength (Lattari et al., 2016; Antunes Faria Vieira et al., 2022). In other words, tCS provides a powerful tool for investigating causal brain-behavior relationships, complementing correlative techniques such as functional EEG-fNIRS neuroimaging (Polanía et al., 2018). Thus, this opinion article aims to highlight the potential of an actual integration of the use of complementary neuroimaging (fNIRS-EEG) and neuromodulation (tCS) techniques with objective behavioral performance metrics (force, velocity) during strength training (Figure 1). They will enable the mapping of hemodynamic (fNIRS) and electrophysiological (EEG) activation and connectivity patterns across various brain regions associated with motor control during strength training in a realistic environment. In addition, recording the effects elicited by tCS through wearable neuroimaging can enhance understanding of the cortical activation profiles engaged during strength training.

## Brain profile monitoring

The aim of strength training is to provide appropriate physiological stimuli to achieve adaptations, including neural adaptations. In addition to an athlete's physical effort, internal load measures refer to the acute responses of the physiological system to a given dose of exercise. Seidel-Marzi and Ragert (2020) proposed the use of neurodiagnostic tools such as EEG or fNIRS in sports performance diagnostics. This is particularly relevant for strength training, where neuroscience research could provide new insights for athlete profiling and a better understanding of how the brain develops during resistance training programs. Only a limited number of studies have examined EEG signals during traditional forms of resistance exercise involving significant muscle mass. The finding that different resistance training protocols involving squat movements provide unique maps of motor related cortical potential indicates the importance of the acute program variables (intensity and volume) to physical development in individuals (Comstock et al., 2011). During bench press movements while recording EEG signals over a single electrode, Engchuan et al. (2017) observed a significant increase in beta and gamma amplitudes as compared to baseline conditions. Unilateral explosive resistance training results in a decrease in motor related cortical amplitudes with concomitant increases in maximal force, rate of force development (RFD), and surface electromyography (Falvo et al., 2010). Herein, reduced cortical drive led to submaximal force generation, which resulted in to the proposal of an enhanced neural economy hypothesis for activation in motor task processing distinguishing expert and novice athletes (Li and Smith, 2022). Furthermore, an EEG study showed that the most fatiguing strength training protocols were associated with the greatest increase in cortical activity (Flanagan et al., 2012). Concerning the type of muscle contraction performed during the squat exercise, the increase in movement control-related areas such as the PMC, SMA, and M1 during the eccentric movement suggests a less efficient process when compared to the concentric movement, but may lead to an increased potential for force capacity production (Kenville et al., 2020). Previous EEG and fNIRS studies suggest that the prefrontal cortex (PFC) plays a critical role in the regulation of cortical motor drive during eccentric movement, particularly when compared to isometric and concentric contractions (Perrey, 2018; Borot et al., 2023). After performing biceps curl exercises until muscle failure, Li et al. (2022) observed a decrease in the efficiency of brain networks in the beta frequency band associated with motor control processes. This suggests that acute bouts of strength exercise modulate the functional characteristics of electrophysiological brain networks in an intensity-dependent manner. Currently, there are few studies on the response to resistance exercise using fNIRS. Exercising with slow unilateral knee-extension movement at 50% 1RM eliciting higher metabolic stress increased oxygenation changes in the contralateral and ipsilateral prefrontal cortex (Formenti et al., 2018), indicating higher cortical resources in untrained participants with fatigue. When examining force levels (0 to 40% 1RM) during the execution of a barbell squat, a positive correlation was found between force and cortical activity in the motor system as determined with fNIRS (Kenville et al., 2017). Taken together, a combination of EEG and fNIRS have the potential to act as effective monitoring tools in exploring the relationships between applied loads and associated brain processing, starting from action selection in the dorsolateral PFC to action sequencing in the SMA, and to action performance in the PMC and M1. In addition, exploring functional changes in the brain following strength training may offer valuable insights into the brain–behavior relationship in both experts and novices. Combining fNIRS with EEG proves beneficial, as EEG can measure neuronal activity at a high temporal resolution for a transient state of the brain. On the other hand, fNIRS can reveal cortical correlates of brain state changes under the neurovascular coupling phenomenon (Sood et al., 2016).

## Brain state regulation

The CNS has the capacity to increase muscle strength by increasing motor unit recruitment. Consequently, changes in the global pattern of cortical activity across multiple brain areas and corticospinal excitability may contribute to improved muscle strength. Neuromodulation technique brought by tCS may be an interesting way to maximize strength training outcomes by modulating the oscillatory brain state in a distributed set of functionally connected brain regions (Seidel-Marzi and Ragert, 2020). Here, the functional state of the brain compared to resting conditions can be referred to the dynamic changes in the concentration of oxygenated blood flow (fNIRS) and a global state of fluctuations in a neural assembly (EEG), often associated to neuromodulation (Harris and Thiele, 2011). Compared to other non-invasive brain stimulation techniques, tCS is currently the most widely used neuromodulation technique in sport and exercise science. By applying a low-intensity direct current (1–2 mA) to the scalp, anodal tCS is generally accepted to increase cortical excitability by lowering the resting membrane threshold of cortical neurons, whereas cathodal tCS decreases neuronal excitability (Polanía et al., 2018). Synaptic plasticity in the motor cortex associated with muscle strength training can be modified by tCS. Despite methodological differences in study design, experimental tasks, tCS parameters and montages in different studies, a number of systematic reviews and meta-analyses over the last 5 years (Alix-Fages et al., 2019; Holgado et al., 2019; Machado et al., 2019; Chinzara et al., 2022; Maudrich et al., 2022) seem to indicate that tCS could be an effective method to increase maximal muscle strength and endurance, but with small amplitude benefits. A plausible explanation for the improvement in muscle strength is that tCS-induced changes in corticospinal excitability increase motor unit recruitment, leading to greater muscle strength during contraction (Lattari et al., 2018). Increased muscle strength is associated with improved force-time characteristics, which enhances the ability to perform common sports skills such as jumping, sprinting and change of direction tasks, and reduce the risk of injury (Suchomel et al., 2016).

In weightlifting, many technical movements require athletes to perform rapid muscle contractions in a short period of time. Athletes with a high RFD, an important index of explosive strength, have a faster rate of muscle contraction and can complete motor tasks more quickly. It has been suggested that RFD is closely related to the recruitment of nerves to motor units per unit time, the frequency of nerve impulses and the type of muscle contraction (Aagaard et al., 1985). Therefore, any improvement in RFD may be due to the increased cortical excitability induced by tCS. However, there are few studies on the enhancement of RFD by tCS (Lu et al., 2021 for the non-dominant limb). A major issue in the field of tCS is the fixed-dose approach, where all individuals receive exactly the same dosage of tDCS, even though we know that individual differences in head and brain anatomy significantly alter how much current enters the brain and where it goes. Real-time assessment of brain activity by EEG and fNIRS and modification of stimulation parameters have been proposed to apply close-loop brain-state dependent brain stimulation (Bergmann et al., 2016). It has been suggested that the effects of tCS may be better understood by examining broader brain networks rather than specific local brain regions (Soleimani et al., 2023). Thus, the assessment of brain state based on EEG-fNIRS monitoring combined with force parameter data requires computational tools that explain the observed intra- and inter-individual variability in tCS responses to resistance exercise and training. Next studies should use machine learning algorithms to analyze data from behavioral outcomes, neuroimaging and physiological characteristics collected from individuals during resistance exercise (Hart et al., 2021). Once important factors for successful tCS outcomes have been identified, a strategy can be proposed to improve the application of tCS by personalizing the dosage (current intensity) for each individual (Albizu et al., 2023). In other words, as with precision medicine, a purely data-driven approach is proposed, using supervised machine learning algorithms to account for individual differences (Kim et al., 2022). Evidence-based medicine is now a widely recognized and established practice with a proven track record (Sackett, 1997). The same is true in sports science, where large amounts of data are collected to individualize coaching as much as possible in the hope of improving performance. Precision training can take into account a large amount of objective data such as movement kinematics, force and cortical signals, which would be processed by computational approaches to help the expert make decisions (Teikari and Pietrusz, 2021) and identify inter-individual and intra-individual variations. Velocity of execution is a reliable indicator of intensity (neuromuscular demand) for programming and monitoring strength training and fatigue management, both on a daily basis and in long-term periodization (González-Badillo and Sánchez-Medina, 2010). In addition to the potential benefits of real-time velocity monitoring during training, the use of real-time brain activation assessment, which neurofeedback training provides (Gong et al., 2021), can track the internal load (brain functional state) exerted on the limbs during strength training, while improving self-regulation (Figure 1). This will allow a more personalized strength training approach to be generated for each individual.

## Conclusion and future directions

Despite important advances in athlete monitoring technologies, there is a limited body of work investigating the effects of strength training on brain function and exercise-induced neuroplasticity. Integrating brain state monitoring seems essential to understanding the interplay between strength training and performance. Future research investigating the neural adaptations to resistance training would be well served by focusing on the use of appropriate portable brain imaging methods to track brain functional state and provide guidance on the tCS dosage. In future experimental research and actual sports training, tCS technology tailored to each individual can be used to increase limb muscle strength in athletes and further increase in overall exercise capacity, which would have extremely practical implications for muscle strength training and the prevention of sports injuries. Such research could have significant implications for optimizing resistance training programmes for athletes and healthy populations, and could lead to a conceptual shift in the way practitioners design and implement resistance training to improve muscular strength.

## Author contributions

SP: Writing—original draft, Writing—review & editing.
